# Comparison of Non-Invasive Individual Monitoring of the Training and Health of Athletes with Commercially Available Wearable Technologies

**DOI:** 10.3389/fphys.2016.00071

**Published:** 2016-03-09

**Authors:** Peter Düking, Andreas Hotho, Hans-Christer Holmberg, Franz Konstantin Fuss, Billy Sperlich

**Affiliations:** ^1^Integrative and Experimental Training Science, Department of Sports Science, Institute for Sport Sciences, Julius-Maximilians University WürzburgWürzburg, Germany; ^2^Data Mining and Information Retrieval Group, Computer Science VI, Artificial Intelligence and Applied Computer Science, Julius-Maximilians University WürzburgWürzburg, Germany; ^3^Department of Health Sciences, Swedish Winter Sports Research Centre, Mid Sweden UniversityÖstersund, Sweden; ^4^School of Sport Sciences, UiT The Arctic University of NorwayTromsø, Norway; ^5^Department of Mechanical and Automotive Engineering, School of Engineering, RMIT UniversityMelbourne, Australia

**Keywords:** wearable technologies, performance parameters, health monitoring, performance monitoring, sports technology

## Abstract

Athletes adapt their training daily to optimize performance, as well as avoid fatigue, overtraining and other undesirable effects on their health. To optimize training load, each athlete must take his/her own personal objective and subjective characteristics into consideration and an increasing number of wearable technologies (wearables) provide convenient monitoring of various parameters. Accordingly, it is important to help athletes decide which parameters are of primary interest and which wearables can monitor these parameters most effectively. Here, we discuss the wearable technologies available for non-invasive monitoring of various parameters concerning an athlete's training and health. On the basis of these considerations, we suggest directions for future development. Furthermore, we propose that a combination of several wearables is most effective for accessing all relevant parameters, disturbing the athlete as little as possible, and optimizing performance and promoting health.

## Introduction

The survey of fitness trends world-wide published in December 2015 (Thompson, 2015) indicates that in 2016 for the first time, wearable technology will become the most popular and leading trend, with the wearable technology market approaching $6 billion dollars. Other trends in fitness, such as body weight training (ranked second in 2016) and high-intensity interval training (ranked sixth) have changed by no more than one place in ranking compared to 2015 (Thompson, 2014). In contrast, in 2015 wearable technology was not ranked at all, probably because it was not even included in the survey.

Adaptation of training is highly individual (Bouchard et al., 1986), depending in part on the balance between exercise and recovery. A suboptimal training load can result in stagnation or de-adaptation, whereas overly intense and/or prolonged training may lead to chronic fatigue, overreaching or overtraining, and negative health effects (Borresen and Lambert, 2009; Buchheit, 2014; Halson, 2014a). In this context, continuous (non-invasive) monitoring of biological and psychological markers might be helpful (Halson, 2014a), and since wearables offer the opportunity to measure different markers conveniently, they provide a promising approach. Wearables are lightweight, sensor-based devices which are worn close to and/or on the surface of the skin, where they detect, analyze, and transmit information concerning several internal and/or external variables to an external device and provide in some cases immediate biofeedback to the athlete. However, the variety of such wearables already available is overwhelming and it is not clear which one(s) may be best for monitoring training and health.

Accordingly, our present aims are threefold: (a) to briefly summarize (non-invasive) parameters that are of potential value in assessing an athlete's training and health; (b) to provide a brief overview of the individual wearables presently available and the parameters they monitor; and (c) to highlight current gaps in our knowledge in order to help direct both future scientific studies and the development of commercial wearables.

## Candidate variables for (non-invasive) monitoring of an athlete's training and health

### Monitoring training status

Monitoring of an athlete's training status must take into consideration the external load applied (i.e., the work completed) in relationship to the individual's response to this load, and a recent review has nicely summarized the various internal and external parameters of potential interest in this context (Halson, 2014a). These parameters and their response to training are highly complex and it is beyond the scope of the present review to discuss them in detail. We simply outline key parameters briefly and refer readers interested in more information to other publications (Borresen and Lambert, 2009; Halson, 2014a).

The external load is usually reflected in parameters such as distance (e.g., when running), velocity (e.g., of running), the duration and frequency of training sessions, etc. (Halson, 2014a). In addition, environmental conditions such as altitude, temperature, and relative humidity can influence the external load (Hargreaves, 2008; Mazzeo, 2008; Drust and Waterhouse, 2010; Maughan et al., 2012; Born et al., 2014) and should therefore be monitored as well.

Among the great variety of relevant internal parameters, some can only be monitored with sophisticated instruments and/or are invasive (e.g., blood analysis) and thereby impractical for daily use (Halson, 2014a). From a practical point of view, monitoring of internal parameters should not only be non-invasive, but also efficiently provide daily simple, yet scientifically trustworthy feedback designed to improve performance and maintain health. Examples include heart rate (HR) during exercise (HRex), as well as recovery (HRR) and variability (HRV) of HR (Achten and Jeukendrup, 2003; Buchheit, 2014; Halson, 2014a).

The HRR is defined as the rate of decline in HR following termination of exercise, which is regulated by the autonomic nervous system and thereby provides information concerning sympathetic and parasympathetic activity (Daanen et al., 2012). In general, the more rapid the HRR, the better the fitness (Daanen et al., 2012; Buchheit, 2014). However, since in trained endurance athletes a period of functional overreaching also appears to be associated with more rapid HRR, this parameter must be evaluated in the context of the training schedule (Aubry et al., 2015).

The HRV, defined as the time that elapses between two heart beats (Achten and Jeukendrup, 2003), can reveal alterations in the autonomous nervous system of the heart (Buchheit, 2014). Even though its applicability is debated (Plews et al., 2013; Halson, 2014a), when assessed longitudinally and at specific time-points (during the night or immediately after waking-up) HRV can help reveal an athlete's training and health status (Plews et al., 2013, 2014; Buchheit, 2014).

In addition to these parameters related to the heart, elevated neuromuscular fatigue (defined as a reduction in force generation due either to central and/or peripheral factors) has been associated with symptoms of overtraining and should be monitored frequently (Fowles, 2006; Cormack et al., 2008; Buchheit, 2014).

Moreover, different lactate thresholds are commonly used to determine an athlete's internal loading and can be used to access the results of training interventions (Bellotti et al., 2013; Halson, 2014a). Consequently, in connection with monitoring an athlete's training status, blood levels of lactate should also be taken into consideration.

### Monitoring health status

Even though the parameters described above are related to those discussed in this section, we highlight here those that provide deeper insight into the training related health status of athletes (Speedy et al., 2001; Halson, 2014b; Saw et al., 2015).

Assessment of hydration status (which is influenced both by the extent of sweating and drinking behavior) is necessary, since dehydration can impair performance and, moreover, is associated with several deleterious health consequences, including heat strokes (Sawka et al., 2007). At the same time, overdrinking can result in hyponatremia and subsequent fatigue, confusion, coma, and even death (Speedy et al., 2001). Consequently, monitoring both fluid loss by sweat and fluid intake is of considerable importance.

When exercising in extreme environments, the athlete's core temperature can exceed 40°C (hyperthermia) or be less than 35° C (hypothermia), which can lead to several kinds of injuries and even threaten life (Armstrong et al., 2007; Fudge et al., 2015).

Ultraviolet (UV) radiation can damage DNA (Cadet et al., 2005) and is a major risk factor for melanoma and other forms of skin cancer (Moehrle, 2008). Consequently, athletes exercising outdoors should monitor their exposure to sunlight, both direct and reflected.

An alteration in the athlete's arterial blood oxygenation (SpO_2_) may explain decrements in performance (Siegler et al., 2007), especially at altitudes where this value is lowered, and may also help to predict acute mountain sickness (Basnyat, 2014).

The quality and quantity of sleep, especially slow-wave sleep during which growth hormones are secreted, are important for recovery, performance, and health and should also be monitored (Halson, 2014b). Impaired sleep disrupts cognitive and immune functions, enhances daytime sleepiness, and reduces overall performance (Leeder et al., 2012; Halson, 2014b).

Subjective parameters, such as mood disturbances or perceived stress and inadequate recovery, can be assessed with different questionnaires that actually appear to provide a more sensitive and consistent evaluation of an athlete's well-being and training load than objective markers (Saw et al., 2015). Accordingly, such questionnaires should be applied with confidence in daily practice (Saw et al., 2015).

## Wearable technologies designed for individual consumers

To evaluate how wearables may assist in monitoring an athlete's training and health, the technologies involved and their abilities to detect specific parameters must be understood.

Several wearables can calculate or estimate body position, movement velocity, distance traveled, and acceleration employing information provided by Global Navigation Satellite Systems (GNSS; such as the Global Positioning System; GPS) (Schutz and Chambaz, 1997; Cummins et al., 2013). With this technology, a good line-of-sight and high-sampling frequency are important for obtaining accurate data (Baranski and Strumillo, 2012; Cummins et al., 2013). Consequently, GNSS measurements do not function indoors or underwater and, moreover, their accuracy may be compromised in densely built-up areas. Inexpensive GPS systems are latent, a problem avoided by high-frequency sampling by professional systems. In contrast, speed tracking appears to be accurate even with inexpensive systems. Position, velocity and distance measured at low-to-moderate velocities (<20 km·h^−1^) by such systems are also reliable, but acceleration data are prone to error and should be interpreted with caution (Cummins et al., 2013; Buchheit et al., 2014).

Accelerometers, which are commonly piezoelectric, piezoresistive, capacitive, or based on strain gauges (Kavanagh and Menz, 2008; Yang and Li, 2012), are used to quantify the distance an athlete covers during training, as well as to evaluate total sleep time and estimate sleep quality, thereby providing an estimate of the quality of sleep (Halson, 2014a). Distance is derived by most accelerometers from the number of steps taken and most count accurately at velocities >67 m·min^−1^ (1.12 m·s^−1^) (Feito et al., 2012), which, however, does not necessarily mean that they measure distance accurately. Accelerometers are reasonably reliable and valid for monitoring the quality and quantity of sleep in certain populations with an accuracy of 80% compared to polysomnography (Leeder et al., 2012; Hausswirth et al., 2014). However, each accelerometer must be fitted securely to prevent motion artifacts (Yang and Hsu, 2010) and accelerometers often fail in detecting the state of wakefulness in sleep periods. Therefore, other methods for the purpose of sleep monitoring are warranted (Sadeh, 2011).

Pulse oximetry exploits the fact that oxyhemoglobin and deoxyhemoglobin absorb near-infrared light maximally at different wavelengths to monitor the oxygen saturation of arterial blood continuously (Chan and Chan, 2013). These sensors are inexpensive, small and simple to use (Chan and Chan, 2013), but prone to potential error due to vasoconstriction, hypovolemia and artifacts caused by excessive movement (Chan and Chan, 2013; Windsor and Rodway, 2014), which limits their usefulness in cold environments and while exercising.

Parameters associated with HR can be monitored with chest belts, photoplethysmography, or various sensors incorporated into clothing. Although chest belts are widely used by athletes, they are experienced as uncomfortable (Buchheit, 2014; Spierer et al., 2015). Photoplethysmography involves a diode on the skin that emits red or near-infrared light that penetrates the underlying tissue and is then reflected back and detected by a photo sensor. This allows assessment of pulse rate with sufficient accuracy at rest, but the error of measurement can be dependent on the photosensitivity of the skin and increases during exercise due to motion artifacts (Schäfer and Vagedes, 2013; Spierer et al., 2015). Consequently, such data should be interpreted with caution. In the case of smart clothing, conducting or metal-coated fibers can be woven into the fabric or conducting inks can be printed onto the garment to monitor HR and associated parameters (Stoppa and Chiolerio, 2014). However, even though promising, only a few studies to date have evaluated the accuracy and reliability of smart clothing (Pandian et al., 2008; Curone et al., 2010) and more are warranted.

To monitor muscle activity by electromyography (EMG), electrodes woven into fabrics have been found to provide values similar to those obtained with traditional surface electrodes (Finni et al., 2007). The drawback of skin electrodes, however, is that

- they must be positioned accurately, preferably “in the midline of the muscle belly between the nearest innervation zone and the myotendinous junction furthest from this zone” (De Luca, 1997), since even small movements away from the innervation zone (e.g., 10% of the muscle length) reduce signal amplitude considerably (Belbasis and Fuss, 2015);
- they must have a tri-polar configuration to allow utilization of “the double differential technique to eliminate the presence of crosstalk” (De Luca, 1997) between different muscles; and
- the signal-force relationship is non-linear and dependend on the number of motor units recruited in the vicinity of the electrode (De Luca, 1997).

Therefore, EMG fabrics designed to assess muscular activity are considered inaccurate. An alternative and promising approach involves incorporation of pressure sensors into compression garments (Belbasis and Fuss, 2015).

To access local muscle oxidative metabolism and to derive lactate thresholds non-invasively, devices which use near-infrared spectroscopy (NIRS) can be employed (Ferrari et al., 2004; Bellotti et al., 2013). These devices are efficient in terms of both time and cost (Bellotti et al., 2013), but are disturbed by adipose tissue (Ferrari et al., 2004) and motion artifacts (Virtanen et al., 2011).

## Overview of commercially available wearable technologies designed for use by athletes

The present discussion here is based on information provided by the manufacturers on their websites. Since the list of available wearables is large and rapidly growing, those described here were chosen if the technology involved was indicated on the website and if they appeared to be the most advanced product of a given manufacturer for a specific purpose. Moreover, we focus solely on wearables that show promise for monitoring the training and health of athletes.

We summarize the wearables chosen (*n* = 36) in Table 1 [wrist-worn devices (*n* = 22)], Table 2 [clothing-based (*n* = 8)], and Table 3 [ear-worn (*n* = 4) and other devices (*n* = 3)], together with the parameters of interest which they monitor and the technology on which they are based. All of these wearables transmit the data they collect to an external device for further analysis and most provide immediate biofeedback to the athlete. So far the accuracy, reliability, or validity of nine devices have been evaluated scientifically (for details please see Tables 1–3).

**Table 1:** **Wrist devices designed to monitor parameters related to the training and health of athletes**.

**Device**	**Training parameters monitored**	**Health parameters monitored**	**Technology employed**	**Additional comments and scientific evaluation**
Polar V800© (Polar Electro, 2015)	D, L, V, El, Alt, HR, HRR, HRV, F	Slt, Slq	GPS, HR chestbelt, additional sensors	Additional Sensors from Polar Electro required. Valid to detect RR intervals with an error of 0.09% and an ICC > 0.99 (Giles et al., 2016)
Microsoft Band 2© (Microsoft, 2015)	D, L, V, HR	UV, Slt, Slq	Accelerometer, ambient light sensor, barometer, capacitive sensor, GPS, GSR, gyroscope, photoplethysmograph, skin temp. sensor, UV sensor	
amiigo© (Amiigo, 2015)	D, L, V, HR, (HRV)	Oxy, Slt, Slq	Accelerometer, pulse oximeter, temp. sensor	
Fitbit Surge© (fitbit Inc., 2015b)	D, L, V, El, HR	Slt, Slq	Accelerometer, altimeter, digital compass, GPS, gyroscope, photoplethysmograph, ambient light sensor	
Suunto Ambit3 Peak (HR)© (Suunto, 2015)	D, L, V, El, Etemp, Alt, HR		GPS, barometer, compass, HR chestbelt	
Withings Pulse Ox© (Withings, 2015)	D, L, El, HR	Oxy, Slt, Slq	Accelerometer, pulse oximeter	The preceding model overestimates sleep time, but had a validity of *r* = 0.92 when compared to a gold standard (Ferguson et al., 2015)
Fitbit charge HR© (fitbit Inc., 2015a)	D, L, El, HR	Slt, Slq	Accelerometer, altimeter, photoplethysmograph	
Garmin vivoactive© (Garmin Ltd., 2015b)	D, L, V, HR	Slt, Slq	Accelerometer, GPS, GLONASS, HR chestbelt	
Garmin vivosmart HR© (Garmin Ltd., 2015c)	D, L, El, HR	Slt, Slq	Accelerometer, altimeter, photoplethysmograph	
Fitbit charge© (fitbit Inc., 2015b)	D, L, El	Slt, Slq	Accelerometer, altimeter	
Garmin Forerunner 910XT© (Garmin Ltd., 2015a)	D, L, V, El, HR		Altimeter, GPS, HR chestbelt	
LG Electronics Lifeband Touch ActivityTracker© (LG Electronics, 2015)	D, L, V, El, HR		Accelerometer, altimeter, HR chestbelt	
Basis Peak© (Basis, 2015)	D, HR	Slt, Slq	Accelerometer, photoplethysmograph, GSR, skin temp. sensor	
Jawbone UP3© (Jawbone, 2015)	D, HR	Slt, Slq	Accelerometer, bioimpedence Sensor	For total sleep time, sleep efficiency, and wake after sleep onset, the preceding model showed good agreement with polyssomnography with a mean difference ± SD of 10.0 ± 20.5 min; −1.9±4.2% and 0.6 ± 14.7 min, respectively (de Zambotti et al., 2015).
Mio Alpha 2© (Mio Global, 2015)	D, L, V, HR		Accelerometer, photoplethysmograph	Its previous model showed significant differences to a reference device for measuring HR at walking, biking (*p* < 0.05) and weight lifting (*p* < 0.01) (Spierer et al., 2015).
Adidas micoach smart run© (adidas Pty. Ltd., 2014)	D, L, V, HR		GPS, photoplethysmograph	
Nike + Sportband© (+Shoe insert) (Nike Inc., 2015a)	D, L, V, HR		HR chestbelt, piezoelectric embed in shoe	
Nike + Sportwatch GPS© (Nike Inc., 2015b)	D, L, V, HR		GPS, HR chestbelt	
Medisana ViFit connect Activity Tracker© (Medisana, 2015)	D, L	Slt, Slq	Accelerometer	
Philips Actiwatch Spectrum Pro© (Philips, 2016)		Slt, Slq, (Sub)	Accelerometer, irradiance sensor, photopic illuminance sensor, Photon Flux sensor	Subjective markers to assess pain only. The sleep accuracy of the preceding modelwas *r* = 0.86 compared to polysomnography (Marino et al., 2013).
Polar Electro Loop©(Polar Electro, 2015)	D, HR	Slt, Slq	Accelerometer, HR chestbelt	
Seraphim Sense Angel Sensor© (Seraphim Sense Ltd., 2014)	D, HR	(Oxy)	Accelerometer, gyroscope, photoplethysmograph, skin temp. sensor	Blood oxygen sensor under development

*Abbreviations: Alt, altitude; D, distance traveled; El, change in elevation; Etemp, environmental temperature; F, neuromuscular fatigue; GLONASS, Global Navigation Satellite System; GPS, General Positioning System; GSR, Galvanic Skin Response; HR, heart rate; HRV, variability of heart rate; HRR, heart rate recovery; ICC, Intraclass correlation coefficient; L, duration of exercise; Oxy, blood oxygenation; Slq, sleep quality; Slt, sleep time; Sub, subjective markers; UV, ultraviolet radiation; V, velocity; (), with restrictions*.

**Table 2:** **Clothing-based wearables designed to monitor parameters related to the training and health of athletes**.

**Device**	**Training parameters monitored**	**Health parameters monitored**	**Technology employed**	**Additional comments and scientific evaluation**
Zephyr Technology Corp. BioHarness™ 3 (Zephyr Technology Corp, 2015)	L, V, HR, HRR, HRV		Accelerometer, GPS, HR sensors woven into fabric	Aims to estimate core temperature by other parameters. HR measurement was reliable (*r* = ~0.91, *p* < 0.01, CV < 7.6) and precise (*r* = 0.61–0.67, *p* < 0.01) when participants performed a walk-jog-run protocol; error increases with increasing velocity (Johnstone et al., 2012).
Carré Technologies Inc. Hexoskin© (Carrè Technologies Inc., 2015)	(D), HR, HRR; HRV		Accelerometer, expansion belts, three point ECG	Not specified if Hexoskin vest or smartphone App measures the parameter duration. The HR measurements showed low CV (%) of < 0.79 ±0.77 and high ICC of >0.96 at different walking speeds (Villar et al., 2015).
Myontec MBody Bike&Run© (Myontec Ltd., 2015)	(D), (L), (V), HR, (F)		EMG, HR chestbelt	EMG technology not further specified, GPS from Smartphone
Ralph Lauren the Polotech Shirt©(Ralph Lauren Media LLC, 2015)	(D), HR		Accelerometer, silver fabrics woven into shirt	
LifeBEAM Smart Hat©(LifeBEAM)	HR		Accelerometer, photoplethysmograph	
LifeBeam Smart Helmet©(LifeBEAM)	HR		Accelerometer, photoplethysmograph	
BSXinsight XR2©(BSXinsight)	Lac		NIRS	

*Abbreviations: CV, coefficient of variation; D, distance traveled; ECG, Electrocardiogram; EMG, Electromyography; F, neuromuscular fatigue; GPS, General Positioning System; HR, heart rate; HRV, variability of heart rate; HRR, heart rate recovery; ICC, Intraclass correlation coefficient; Lac, lactate threshold; L, duration of exercise; Slq, sleep quality; Slt, sleep time; V, velocity; (), with restrictions*.

**Table 3:** **Ear devices and other wearables designed to monitor parameters related to the training and health of athletes**.

**Device**	**Training parameters monitored**	**Health parameters monitored**	**Technology employed**	**Additional comments and scientific evaluation**
**EAR DEVICES**				
FreeWavz© (FreeWavz)	L, V, HR	Oxy	Accelerometer, pulse oximeter	Functions as a music player
Cosinuss One© (cosinuss, 2015)	(D), HR, HRV	(Oxy), Btemp	Photoplethysmograph, temp. sensor	Blood oxygenation sensor under development
Lumafit© (Lumafit, 2015)	D, HR, HRR		Accelerometer, photoplethysmograph	Duration via a Smartphone App
Bragi The Dash© (Bragi, 2015)	D,HR	Oxy	Accelerometer, gyroscope, magnetometer, pulse oximeter	Functions as a music player
**OTHER DEVICES**				
BodyMedia Fit© (BodyMedia Inc, 2014)		Slt, Slq	Accelerometer, GSR, heat flux, skin temp.	Worn on the upper arm, designed to measure energy expenditure
Catapult Optimeye S5© (Catapult innovations, 2015)	D, L, V		Accelerometer, GPS, GLONASS, gyroscope	Worn in a specially design vest. The catapult model is reliable (TEM: 1.3%) and valid for measuring distance with compromised data when the sampling frequency is low (i.e., 1–5 Hz), athletes speed increases and/or at short distances (Jennings et al., 2010; Johnston et al., 2014). The accuracy of accessing velocity has a CV of 3.1–11.3%for the 10 Hz unit (Varley et al., 2012)
Misfit Shine© Misfit Wearables	L	Slt, Slq	Accelerometer	Can be worn anywhere on the body

*Abbreviations: Btemp, body temperature; D, distance traveled; GPS, General Positioning System; GSR, Galvanic Skin Response; GLONASS, Global Navigation Satellite System; HR, heart rate; HRV, variability of heart rate; HRR, heart rate recovery; L, duration of exercise; Oxy, blood oxygenation; Slq, sleep quality; Slt, sleep time; TEM, typical error of measurement; V, velocity; (), with restrictions*.

### Wrist-worn devices

Most wrist-worn devices employ accelerometers (*n* = 16), gyroscopes (*n* = 3), GNSS (*n* = 8), (barometric) altimeters (*n* = 8), photoplethysmography (*n* = 8), additional chest belts (*n* = 8), sensors of skin temperature (*n* = 4), pulse oximeters (*n* = 2) and/or sensors of UV light (*n* = 1) to monitor duration of activity (*n* = 21), distance (*n* = 17), and velocity (*n* = 12) of an athlete's locomotion, change in elevation (*n* = 10), environmental temperature (*n* = 1), altitude (*n* = 2), HR (*n* = 19), HRV (*n* = 2), neuromuscular fatigue (*n* = 1), UV radiation (*n* = 1), SpO_2_ (*n* = 3), sleep quality and quantity (*n* = 14), and subjective markers (*n* = 1). However, HR recovery, humidity, hydration status, lactate thresholds and body temperature are not assessed. Furthermore, the only wearable that accesses subjective markers focuses on pain, but no other factors related to the training and health status of athletes.

The previous model of the Philips Actiwatch Spectrum Pro© (Philips Respironics, Murrysville, PA, USA) showed high accuracy to detect sleep compared to polysomnography, however, its ability to detect wakefulness is low (Marino et al., 2013).

The preceding model of the Withings Pulse Ox© (Withings SA, Issy-les-Moulineaux, France) overestimates sleep time with a validity of *r* = 0.92 compared to polysomnography (Ferguson et al., 2015).

The Polar V800 (Polar Electro, Kempele, Finland) is valid to detect RR intervals with an error of 0.09% and an intra-class correlation coefficient of >0.99 (Giles et al., 2016).

The preceding model of the Mio Alpha 2© gave HR values while walking, weight lifting, and biking that differed significantly from those obtained with a reference device and this model appears to be prone to motion artifacts (Spierer et al., 2015).

The Jawbone UP© (the model preceding the Jawbone UP3 we describe) was validated for measurement of total sleep time and time-point of awakening after sleep onset and showed good agreement with polysomnography (de Zambotti et al., 2015).

To the best of our knowledge all other devices have not been evaluated scientifically and, accordingly, the data they provide should be interpreted with considerable caution.

### Devices incorporated into clothing

Specially designed (“smart”) clothing, ranging from shirts, shorts, hats/helmets to socks, can monitor several internal and external parameters of relevance to athletes. Most “smart” clothing currently available utilizes accelerometers (*n* = 5), electrocardiography (*n* = 1), additional chest belts (*n* = 1), photoplethysmography (*n* = 2), and/or conducting fibers woven into the fabric (*n* = 2) to measure HR (*n* = 7), HR recovery (*n* = 2), HR variability (*n* = 2), neuromuscular fatigue (by EMG, *n* = 1), and lactate threshold (by NIRS, *n* = 1). To assess the external parameters duration (*n* = 3), distance (*n* = 2), velocity (*n* =2), and change in elevation (*n* = 1), “smart” clothes (with exception of the Zephyr BioHarness™ 3) rely on the data transmitted by the companion smartphone. The BioHarness™ 3 (Zephyr Technology Corp, Annapolis, USA) also aims to derive body temperature from the parameters it assesses (Zephyr Technology Corp, 2015). To date, environmental temperature, humidity, UV radiation, SpO_2_, hydration status, quantity and quality of sleep, and subjective factors have been neglected by designers of “smart” clothing. Furthermore, no “smart” clothing presently available can provide immediate biofeedback to the athlete without the involvement of an external device.

The Hexoskin© vest (Carré Technologies Inc., Montreal, Québec Canada) provides reliable detection of an athlete's HR when lying, sitting, standing or walking slowly (%CV < 0.79 ± 0.77; ICC > 0.96; Villar et al., 2015). However, measurement of the other parameters relevant to the training and health of athletes has not yet been validated, least of all when training.

The BioHarness™ 3 has an acceptable level of validity and reliability for HR (*r* = ~0.91, *p* < 0.01; %CV < 7.6), but increasing errors at higher velocity (Johnstone et al., 2012). Measurement of HRR and HRV by this device has not been evaluated scientifically. Since, at least to our knowledge, no other form of “smart” clothing has yet been evaluated scientifically, the data they provide should be interpreted with due caution.

### Ear-worn devices

Devices worn as an earplug (*n* = 3) or around the auricle (*n* = 1) use accelerometers (*n* = 3), pulse oximeters (*n* = 2), photoplethysmography (*n* = 2), temperature sensors (*n* = 1), gyroscopes, and magnetometers (*n* = 1) to assess duration (*n* = 3), distance covered by the athlete while training (*n* = 1), velocity (*n* = 1), HR (*n* = 4), HRV (*n* = 1), HRR (*n* = 1), SpO_2_ (*n* = 3), and body temperature (*n* = 1). However, it should be noted that such devices measure variations in pulse rate rather than HRV directly (Schäfer and Vagedes, 2013). Parameters such as change in elevation, environmental temperature, humidity, altitude, neuromuscular fatigue, UV radiation, hydration status, lactate thresholds, quantity and quality of sleep, as well as subjective markers cannot yet be monitored by ear-worn wearables.

### Other devices

A number of other devices are designed to be worn on specific parts of the body. The BodyMedia Fit© armbands (BodyMedia Inc., Pittsburgh, PA, USA), designed to measure energy turnover, are worn on the upper arm and use an accelerometer in combination with sensors of sweating (the galvanic skin response, GSR), heat flux and skin temperature to recognize motion and thereby monitor an athlete's quantity and quality of sleep, in addition to other parameters not directly relevant to training and health (BodyMedia Inc, 2014). This system calculates the energy expenditure associated with various physical activities reliably (Lee et al., 2014).

The Misfit Shine© (Misfit Wearables, Burlingame, CA, USA) monitors distance, as well as the quantity and quality of sleep with an accelerometer. It can be worn anywhere on the body and transfers the data collected solely to an external device (Wearables). The sleep time measured by Misfit Shine© correlates well to that provided by a reference device, although with some overestimation (Ferguson et al., 2015). No other parameters are monitored.

The Catapult Optimeye S5© (Catapult Innovations, Melbourne, VIC, Australia) utilizes GPS, GLONASS (the Russian equivalent of the American GPS), an accelerometer and gyroscope to track duration, distance, and velocity. This device is worn in a specially designed vest below the neck (Catapult innovations, 2015). The previous model was shown to be valid for determining distance (Johnston et al., 2014), as well as sensitive for assessing velocity (Varley et al., 2012). However, the reliability of these devices is less at short distances or with increasing speed and appears to depend on the sampling frequency (Jennings et al., 2010).

## Recommendations concerning wearables for athletes

As indicated above, most of the wearables currently available have not yet been evaluated scientifically, even though evaluation of their reliability, validity and accuracy at the very least, particularly in connection with training, is critical for athletes to be able to use them with confidence.

In addition to movement artifacts, the frequency of sampling by a wearable may compromise the quality of the data collected. Although a low frequency may be adequate when the athlete is at rest, a higher frequency is required during exercise when parameter values alter relatively quickly. Scientific evaluation can help determine a sampling frequency that provides sufficient accurate feedback to the athlete, while still being manageable by the storing and processing capacities of the wearable.

In light of these considerations, we strongly advice manufacturers of wearables to arrange for independent scientific evaluation of their products and to base future development on such information.

Most wearables focus on monitoring duration (*n* = 28), distance (*n* = 22), velocity (*n* = 16) and sometimes changes in elevation (*n* = 11), in combination with the HR (*n* = 30) and sleep (*n* = 16). Environmental temperature (*n* = 1), altitude (*n* = 2), HRV (*n* = 5), HRR (*n* = 3), neuromuscular fatigue (*n* = 2), lactate thresholds (*n* = 1), UV radiation (*n* = 1), and body temperature (*n* = 1) can be assessed by a few; whereas humidity, hydration status, and subjective factors relevant to training and health are completely neglected by all. Therefore, we strongly advise manufacturers to develop devices capable of monitoring such parameters as well.

Measurement of environmental parameters such as temperature, altitude, UV radiation, and humidity is relatively straightforward and should become standard in future wearables.

HRR can be derived from HR, which many of the wearables discussed here can monitor, so that all that is required in this case is additional software. HRV can be derived from variations in pulse rate at rest, so that wearables might focus on this parameter, which is probably easier to access.

Neuromuscular fatigue (at least for the legs) as reflected in a countermovement jump (Cormack et al., 2008) while wearing a low-cost and pressure-sensitive insole (Tan et al., 2015) or a compression garment equipped with pressure sensors could be assessed (Belbasis and Fuss, 2015).

Only one wearable presently available to consumers is able to measure lactate thresholds (via NIRS), and this device focuses on the muscles of the lower leg only. To obtain a more complete picture, it would be desirable to apply NIRS to other groups of muscles as well.

An athlete's hydration status is influenced by how much he/she sweats and this should to be assessable by wearables. The textile sensors developed in connection with the BIOTEX project are already able to determine this, as well as the level of sodium in the sweat (Coyle et al., 2010).

Another sensor designed to assess hydration status appears to be close to being introduced onto the market. In 2015 a transdermal sensor that analyses electrolytes in sweat was developed by the University of Strathclyde. This device provides real-time analysis of fluid loss, with feedback to the user via smart phone, to encourage proper rehydration (University of Strathclyde, 2015).

To the best of our knowledge, no wearables incorporate assessment of subjective factors associated with training and health. This is surprising, since subjective measures have been shown to be superior to objective markers in this context (Saw et al., 2015) and it should be simple to incorporate questionnaires into the software of external devices. One of the subjective variables most commonly monitored in connection with studies on exercise physiology is the “rating of perceived exertion” (RPE) first described by Borg (Borg, 1970), which has proven to be highly sensitive for evaluating general fatigue from different types of exercise (Grant et al., 1999). It is remarkable that the RPE, an easy accessible variable, has not yet been incorporated into any wearable.

However, since monitoring certain parameters requires placement of a device at a specific anatomical location, monitoring of all parameters of interest may not be achievable with a single wearable. For example, body temperature is measured ideally with an ear-worn device, hydration status based on the extent of sweating is best evaluated by clothing which covers as much of the skin as possible; and it is preferable to monitor sleep with a wearable worn on the wrist. At the same time, bearing several different wearables simultaneously might be cumbersome.

Luckily, not all parameters appear to be equally relevant at any given time. For example, HRV is measured ideally during the nighttime or upon waking up (Buchheit, 2014), whereas the hydration status of an athlete is of primary concern before, during and after exercise. Consequently, in order to assess all relevant parameters at the time when they are of most interest while disturbing the athlete as little as possible, it might be of interest to develop a commercial monitoring system composed of different wearables placed at their ideal locations.

Nonetheless, even when wearables provide appropriate and reliable daily feedback to the athlete, these data are interpreted either by the athlete him/herself and/or the algorithm in the manufacturer's software, rather than by professionals, which may lead to inappropriate adjustments in training. Furthermore, the wearables presently available are restricted to non-invasive parameters and without at least minimal assessment of invasive parameters, such as the levels of substances in capillary blood and saliva (e.g., cortisol or immunoglobulin A, creatine kinase, urea, and other markers of immune function and muscle damage), the information provided may be incomplete. Thus, manufacturers of wearables should focus on convenient and rapid measurement of such parameters as well.

Wearables are becoming less and less expensive and more and more athletes are monitoring their health and training and storing this information centrally. This information can be used not only for optimizing individual training, but also for more general (and global) analysis employing data mining, machine learning, and statistical methods. For example, weak signals concerning patterns of movement such as walking, running, climbing stairs, sitting, etc., from a simple sensor could be uploaded into the cloud for analysis. Moreover, typical patterns of behavior in athletic scenarios, such as training camps in and off season, could be identified. The resulting insights could then be used in the development of wearables that provide even better assessment of training and health. However, uploading intimate personal data to unsecure servers and potential commercial use of such data are of considerable concern with respect to individual privacy.

## Conclusion

In summary, wearables designed to monitor a variety of non-invasive parameters must be evaluated scientifically before these can be confidently employed to assess the training and health status of athletes. Otherwise, the athlete should be skeptical about the usefulness of a wearable in practice.

Furthermore, certain important parameters are completely ignored by today's wearables, even though effective approaches to measuring these parameters are already available. Since monitoring training and health is rather complex, requiring that several parameters be evaluated at different times of the day and on different parts of the body, we propose that a combination of wearables is needed to obtain an overall picture while disturbing the athlete as little as possible. This would help athletes to improve their performance and reduce the risk of injuries from exercise and training.

## Author contributions

All authors listed, have made substantial, direct and intellectual contribution to the work, and approved it for publication.

### Conflict of interest statement

The authors declare that the research was conducted in the absence of any commercial or financial relationships that could be construed as a potential conflict of interest.
